# Translation, cultural adaptation, and validation of the EORTC QLQ-CR29 questionnaire in Serbian patients with colorectal cancer

**DOI:** 10.1017/S1478951525100904

**Published:** 2025-10-20

**Authors:** Vladimir Nikolić, Ljiljana Markovic-Denic, Velimir Markovic, Aleksandar Radovanovic, Djordje Nektarijevic, Stefan Kmezic, Jelena Djokic Kovac, Andrija Antic

**Affiliations:** 1 University of Belgrade, Faculty of Medicine, Institute of Epidemiology, Belgrade, Serbia; 2 Department of Gastrointestinal Surgery, University Clinical Center of Serbia, Clinic of Digestive Surgery, Belgrade, Serbia; 3 Faculty of Medicine, University of Belgrade, Belgrade, Serbia; 4 University Clinical Center of Serbia, Institute of Radiology, Belgrade, Serbia

**Keywords:** Colorectal cancer, quality of life, Serbia, validation, psychometrics

## Abstract

**Objectives:**

This study aimed to translate, culturally adapt, and validate the European Organisation for Research and Treatment of Cancer Quality of Life Questionnaire for Colorectal Cancer for Serbian patients.

**Methods:**

The prospective cohort study was conducted at the Clinic for Digestive Surgery, University Clinical Center of Serbia, and included 150 Serbian-speaking colorectal adenocarcinoma patients undergoing colorectal surgery. The translation process involved rigorous forward and backward translations, pilot testing with patients, and statistical analysis for psychometric validation, including internal consistency, reliability, convergent and discriminant validity, concurrent validity, and known-groups validity.

**Results:**

Results showed good internal consistency across most scales (Cronbach’s alpha values ranging from 0.769 to 0.855), with excellent split-half reliability (0.872). Convergent and discriminant validity analyses confirmed the questionnaire’s capacity to measure constructs it was theoretically related. The significant correlations were observed between corresponding scales and items of EORTC QLQ-C30 and EORTC QLQ-CR29 questionnaires. Known-groups analysis demonstrated the tool’s ability to distinguish between patient groups based on tumor location, stoma presence, and neoadjuvant therapy.

**Significance of results:**

The Serbian version of the EORTC QLQ-CR29 is a reliable and valid instrument for assessing the quality of life in Serbian colorectal cancer patients, reflecting its potential for widespread clinical application.

## Introduction

Colorectal cancer is a major global health issue, with over 1.9 million new cases in 2020, ranking third in men and second in women worldwide (Sung et al. 2021). The highest incidences were observed in Europe, Australia, New Zealand, and North America, with projections estimating 3.2 million new cases and 1.6 million deaths by 2040 (Morgan et al. 2023). In 2021, Serbia reported 5,174 new cases, representing 12.4% of all cancers and making it the second most common cancer in the country. It affects more men than women, with age-adjusted rates of 44.3 and 24.7 per 100,000, respectively, and is most common in those over 75 years old (Cancer Registry 2023). The incidence of colorectal cancer has been increasing over the last 30 years in Serbia and other Balkan countries (Todorovic et al. 2023).

Assessing quality of life (QoL) is essential in cancer management, offering insights into the patient’s experience of disease and treatment effects, covering physical, emotional, cognitive, and social aspects (Aaronson et al. 1993; Qedair et al. 2022; Parsons et al. 2019). The EORTC QLQ-CR29, a module for colorectal cancer derived from the QLQ-CR38, is designed to measure health-related QoL, addressing specific symptoms, treatment side effects, body image, sexuality, and future perspectives (Ganesh et al. 2016; Gujral et al. 2007; Sprangers et al. 1999; Whistance et al. 2009). The validation of this instrument among different populations is crucial for its application, ensuring the relevance and sensitivity to the specific quality of life impacts of colorectal cancer and its treatment (Bachri et al. 2023; Ihn et al. 2015; Kishore et al. 2021; Lin et al. 2017; Sanna et al. 2017). The Serbian adaptation of the FACT-C questionnaire highlights the importance of cultural and linguistic adjustments for such tools (Ilić-Živojinović et al. 2022).

The aim of our study was to translate, culturally adapt, and psychometrically validate the EORTC QLQ-CR29 questionnaire for Serbian colorectal cancer patients.

## Methods

The prospective cohort study was conducted at the Clinic for Digestive Surgery, University Clinical Center of Serbia. It is the largest university center in our country, covering around 1.6 million residents of the capital city of Belgrade, and part of Serbia. The study population consisted of patients admitted to the clinic due to colorectal carcinoma between May 2022 and February 2023.

The study inclusion criteria were: patients aged 18 and older who were diagnosed with colorectal adenocarcinoma and were candidates for colorectal surgery. All patients were Serbian native speakers. Patients with a stoma due to the presence of obstructive colorectal cancer were also included in the study.

The exclusion criteria for the study were: patients younger than 18 years, presence of inflammatory bowel disease, colorectal surgeries for diverticulitis and benign polyps, ischemic colitis, hereditary colorectal neoplasia, recurrent adenocarcinoma, patients with present metastases, patients who were unable or refused to give informed consent, as well as patients with whom it was not possible to communicate due to cognitive impairment.

### Questionnaires

*EORTC QLQ-C30 Version 3.0*: A 30-item questionnaire developed to assess the quality of life of cancer patients. It includes 5 functional scales (physical, role, emotional, cognitive, and social functioning), 3 symptom scales (fatigue, nausea/vomiting, and pain), a global health/QoL scale, and 6 single items (dyspnea, insomnia, appetite loss, constipation, diarrhea, and financial difficulties). Items 29 and 30 have 7 possible responses, while the rest have 4 (ranging from 1 [not at all] to 4 [very much]). For all scales, firstly, the raw score was calculated as the mean of the component items. The score for the functional scales score was calculated according to the formula: score = (1 − (RS − 1)/range) × 100 and for the symptom scales or items and global health status/QoL according to the formula: score = ([RS − 1]/range) × 100. All of the scales and single items range in score 0–100 (Aaronson et al. 1993; Fayers et al. 2001; Velikova et al. 2012).

*EORTC QLQ-CR29*: A disease-specific module for colorectal cancer patients addressing symptoms, side effects, body image, sexual functioning, and future perspective. It consists of 29 questions covering 5 functional domains (Appearance, Anxiety, Weight, and Sexual Interest in Men and Women) and 18 symptom-related domains. It has 4 multi-item scales (urinary frequency, blood and mucus in stool, stool frequency, and body image) and 19 single-items. Separate questions are provided for patients with and without a stoma, and questions for men and women regarding sexuality. Most questions pertain to the period of the last 7 days, except for sexuality, which covers 4 weeks. For all scales, firstly, the raw score was calculated as the mean of the component items. For single-items raw score is the value of that item. The score for the functional scales score was calculated according to the formula: score = (1 − (RS − 1)/range) × 100 and for the symptom scales or items and global health status/QoL according to the formula: score = ([RS − 1]/range) × 100. All of the scales and single items range in score 0–100. Higher functional scores indicate better functioning and higher symptom scores indicate more problems (Whistance et al. 2009).

### Translation and cultural adaptation of the EORTC QLQ-CR29

The translation process was carried out according to the recommended steps (EORTC Quality of Life Group 2017), starting with 2 forward translations by Serbian native speakers fluent in English. These versions were then reconciled by a third party, combining the best elements of both. Subsequently, this reconciled version underwent back-translation into English by 2 translators, highly proficient in English. Then back translation report was compiled detailing the translation steps, choices made, and comments, which was reviewed by the EORTC translation unit (TU). Upon addressing any feedback, an external proofreader, selected by the TU, reviewed the preliminary translation. The feedback received was thoroughly discussed until a consensus was reached. Pilot-testing with 15 patients confirmed the translation’s comprehensibility, with no significant issues reported. After thorough review and consensus, the TU approved the final translation, ensuring it met EORTC standards and the validation study’s objectives.

### Statistical analysis

Data are presented as mean ± SD for continuous variables and number (percentage) for categorical variables. For the comparison of CR29 scores and items between groups independent T test or Mann-Whitney test were used where appropriate based on the normality of distribution. The *p*-values less than 0.05 were considered statistically significant. At scale level, floor and ceiling effects were considered to be present if more than 15% of respondents achieved, respectively, the lowest or the highest possible score (Terwee et al. 2007). The statistical analyses were performed using SPSS version 23.0 software (SPSS Inc., Chicago, IL, USA) and R 4.3.2. (R Core Team (2023). R: A language and environment for statistical computing. R Foundation for Statistical Computing, Vienna, Austria. URL https://www.R-project.org/.).

#### Construct validity

For construct validity Cronbach’s alpha coefficients were calculated to assess internal consistency. A value above 0.70 was considered acceptable (Cronbach 1951). Construct validity involved assessing convergent and discriminant validity using the multitrait-multimethod (MTMM) analysis (Lowe and Ryan-Wenger 1992). Convergent Validity was determined by examining the correlations between items and scales within the EORTC QLQ-CR29 that should be related. Pearson’s correlation coefficients were used to assess these relationships, with coefficients of 0.40 or higher indicating good convergent validity. Discriminant validity was assessed by investigating the extent to which scales and items intended to measure different constructs demonstrated low correlations with each other. Low correlations (r < 0.30) between constructs that are theoretically distinct, demonstrating the questionnaire’s ability to differentiate various aspects of QOL.

#### Reliability

To estimate the reliability of the CR29 questionnaire, a split-half reliability analysis was conducted (de Vet et al. 2017). This method evaluates the consistency of responses across 2 equivalent halves of the test. The correlation between the 2 halves’ scores was determined using the Pearson correlation coefficient as a preliminary step in the reliability estimation. This correlation served as the basis for the split-half reliability calculation, which was then adjusted for the full test length using the Spearman-Brown prophecy formula (de Vet et al. 2017).

#### Concurrent validity

The concurrent validity of the EORTC QLQ-CR29 questionnaire was evaluated by comparing it with the EORTC QLQ-C30. The analysis focused on calculating Pearson’s correlation coefficients between corresponding scales and items of the QLQ-CR29 and QLQ-C30 questionnaires (Lin and Yao 2014).

#### Known-groups validity

Clinical validity was assessed using the known-group method (Davidson 2014). Groups for comparison were based on the tumor location (colon and rectum), presence of a stoma and neoadjuvant therapy.

The study was conducted in accordance with the principles of the Declaration of Helsinki (2000 revision of Edinburgh). Our study was approved by the Ethics Committee of the University Clinical Center of Serbia (Number 808/5). All patients signed the informed consent to participate in the study before filling out the questionnaires.

## Results

A total of 153 patients were approached for participation in the study: Of this, 2 declined, and communication was not possible with 1, resulting in 150 patients being included. The average age of included patients was 64.7 years, with a slight male predominance (53.3%). Sociodemographic and clinical characteristics of patients are presented in Table 1.

**Table 1.** Sociodemographic and clinical characteristics of patients

	n (%)
Total	150 (100%)
Age (mean ± SD)	64.7 ± 10.8 (median 67 years)
Gender	
Male	80 (53.3)
Female	70 (46.7)
BMI (mean ± SD)	25.99 ± 4.75
Education	
Elementary school	14 (9.3)
High school	101 (67.3)
Faculty	35 (23.3)
Marital status	
Married	111 (74.0)
Divorced	12 (8.0)
Unmarried	7 (4.7)
Widowed	20 (13.3)
Employment	
Unemployed	8 (5.3)
Employed	47 (31.3)
Pension	95 (63.3)
Smoking status	
Nonsmoker	64 (42.7)
Previous smoker	54 (36.0)
Current smoker	32 (21.3)
Alcohol consumption	
No	76 (50.7)
Rarely	14 (9.3)
Few times a month	22 (14.7)
At least once a week	24 (16.0)
Daily	14 (9.3)
Charlson comorbidity index (mean ± SD)	4.4 ± 1.3
Mild (CCI scores of 1–2)	13 (8.7)
Moderate (CCI scores of 3–4)	67 (44.7)
Severe (CCI scores ≥ 5)	70 (46.7)
ASA score	
I	7 (4.7)
II	82 (54.7)
III	57 (38.0)
IV	4 (2.7)
Tumor localization	
Colon	76 (50.6)
Rectum	73 (48.7)
Both	1 (0.7)
Neoadjuvant radiotherapy	37 (24.7)
Neoadjuvant chemotherapy	39 (26.0)
Stoma	
No	134 (89.3)
Yes	16 (10.7)

SD – standard deviation, BMI – body mass index, CCI – Charlson Comorbidity Index, ASA – American Society of Anesthesiologists

Among the symptom scales, urinary frequency, blood and mucus in stool and stool frequency scales demonstrated good internal consistency with Cronbach’s alpha values of 0.769, 0.802 and 0.810, respectively. Furthermore, regarding functional scales, the body image scale exhibited a Cronbach’s alpha value of 0.855, indicating excellent internal consistency. The CR-29 questionnaire structure with internal consistency is presented in Table 2.

**Table 2.** EORTC QLQ-CR29 questionnaire structure and internal consistency

Scaling/single-item name	n	Item numbers	Mean	SD	Range	% floor	% ceiling	α
Urinary frequency	150	31, 32	34.4	24.0	0–100	17.3	1.3	0.769
Urinary incontinence	150	33	8.2	18.9	0–100	81.3	0.7	
Dysuria	150	34	4.9	15.2	0–100	88.7	0.7	
Abdominal pain	150	35	22.2	28.3	0–100	54.0	4.0	
Buttock pain	150	36	16.7	23.7	0–100	61.3	1.3	
Bloating	150	37	26.0	26.2	0–100	42.0	2.0	
Blood and mucus in stool	150	38, 39	23.8	25.6	0–100	37.3	1.3	0.802
Dry mouth	150	40	24.2	25.3	0–100	44.0	2.0	
Hair loss	150	41	6.4	18.0	0–100	86.7	0.7	
Taste	150	42	5.8	16.7	0–100	87.3	0.7	
Flatulence	150	49	21.3	29.2	0–100	57.3	5.3	
Fecal incontinence	150	50	11.8	23.5	0–100	75.3	2.7	
Sore skin	150	51	11.3	19.2	0–100	70.7	0.7	
Stool frequency	150	52, 53	15.5	22.7	0–100	56.7	1.3	0.810
Embarrassment	150	54	12.9	25.5	0–100	75.3	3.3	
Stoma care problems	16	55	18.7	21.0	0–67	50.0	6.3	
Impotence	80	57	28.7	29.4	0–100	42.5	3.8	
Dyspareunia	70	59	10.5	22.4	0–100	77.1	2.9	
Anxiety	150	43	44.9	30.6	0–100	19.3	11.3	
Weight	150	44	71.1	29.1	0–100	4.7	40.7	
Body image	150	45–47	79.5	24.5	0–100	2.7	42.0	0.855
Sexual interest (men)	80	56	41.7	28.3	0–100	9.3	4.7	
Sexual interest (women)	70	58	9.0	17.9	0–67	36.0	2.0	

SD – standard deviation, % floor – proportion of patients who scored at the lowest possible score, % ceiling – proportion of patients who scored at the highest possible score, α – Cronbach’s alpha.

The analysis revealed a split-half reliability coefficient of 0.872, signifying an excellent level of reliability of EORTC QLQ-CR29 questionnaire.

Overall, the EORTC QLQ-CR29 questionnaire shows good convergent validity for all examined scales. Divergent validity is also generally supported, with most scales showing low correlations with unrelated constructs (Table 3).

**Table 3.** Convergent and divergent validity of EORTC QLQ-CR29 questionnaire

	Total sample (n = 150)			Without stoma^a^ (n = 134)			With stoma^b^ (n = 16)		
Scales	Convergent	Divergent	α	Convergent	Divergent	α	Convergent	Divergent	α
Urinary frequency	0.627–0.917	0.004–0.268	0.769	0.619–0.905	0.057–0.254	0.764	0.705–0.908	0.000–0.330	0.820
Blood/mucus in stool	0.672–0.924	0.010–0.337	0.802	0.712–0.934	0.019–0.349	0.828	0.510–0.972	0.037–0.495	0.654
Stool frequency	0.699–0.939	0.004–0.337	0.810	0.781–0.953	0.001–0.349	0.871	0.237–0.967	0.000–0.576	0.225
Body image	0.597–0.907	0.007–0.268	0.855	0.590–0.906	0.001–0.255	0.853	0.668–0.911	0.012–0.576	0.872

a Pearson’s correlation coefficient; ^b^Spearman’s correlation coefficient; α – Cronbach’s alpha.

The correlations presented in Table 4 between CR-29 and QLQ-C30 scales/items demonstrate the concurrent validity of the CR-29 questionnaire. Body image positively correlated with most functional scales. Conversely, scales such as urinary frequency, blood and mucus in stool, and stool frequency showed negative correlations with functional scales but positive correlations with symptom scales like fatigue.

**Table 4.** Correlation between EORTC QLQ-CR29 and C30 questionnaires

	QLQ-C30														
	Functional scales						Symptom scales/items								
CR-29 scales	QL	PF	RF	EF	CF	SF	FA	NV	PA	DY	SL	AP	CO	DI	FI
Urinary frequency	−0.168*	−0.217**	−0.288**	−0.094	−0.162*	−0.056	0.310**	0.065	0.176*	−0.056	0.096	0.171*	0.129	−0.015	0.076
Blood and mucus in stool	−0.211**	−0.034	−0.107	−0.222**	−0.183	−0.210**	0.207*	0.112	0.298**	0.198*	0.164*	−0.006	−0.104	0.170*	0.185
Stool frequency	−0.207*	−0.127	−0.237**	−0.109	−0.177*	−0.274**	0.232**	0.272**	0.198*	0.211**	−0.055	0.129	0.025	0.463**	0.167*
Body image	0.337**	0.291**	0.446**	0.274**	0.308**	0.449**	−0.398**	−0.207*	−0.296**	−0.279*	−0.079	−0.322**	−0.104	−0.163*	−0.193*
CR-29 single items															
Urinary incontinence	−0.100	−0.175*	−0.170*	−0.002	−0.154	−0.064	0.166	−0.082	0.017	−0.089	0.041	0.067	−0.015	0.146	0.024
Dysuria	0.014	−0.106	−0.145	−0.033	−0.208*	−0.106	0.101	0.071	0.246**	0.253**	0.139	0.119	0.126	−0.081	0.120
Abdominal pain	−0.303**	−0.184*	−0.272**	−0.277**	−0.288**	−0.198*	0.353**	0.379**	0.663**	0.145	0.229**	0.335**	0.369**	0.042	0.175*
Buttock pain	−0.230**	−0.167*	−0.266**	−0.265**	−0.322**	−0.203*	0.256**	0.197*	0.440**	0.104	0.135	0.328**	0.230**	0.052	0.278**
Bloating	−0.238**	0.014	−0.050	−0.246**	−0.014	−0.192*	0.056	0.168*	0.204*	0.072	0.037	0.057	0.176*	0.091	0.190*
Dry mouth	−0.111	−0.222**	−0.343**	−0.190*	−0.297**	−0.206*	−0.330**	0.077	0.214**	0.273**	0.279**	0.102	0.093	0.046	0.096
Hair loss	−0.130	−0.201*	−0.203*	0.157	0.124	−0.215**	0.132	0.070	0.222**	0.103	0.013	0.064	0.083	−0.098	0.061
Taste	−0.137	−0.278**	−0.201*	−0.074	−0.115	−0.058	0.241**	0.146	0.262**	0.182*	0.103	0.237**	0.302**	0.119	0.053
Flatulence	−0.262**	−0.010	−0.076	−0.196*	−0.066	−0.105	0.095	0.180*	0.160*	0.100	0.135	0.097	0.095	0.118	0.235**
Fecal incontinence	−0.157	0.020	−0.205*	−0.171*	−0.074	−0.142	0.189*	0.079	0.226**	0.172*	0.069	0.191*	−0.010	0.186*	0.255**
Sore skin	−0.160	0.072	0.007	−0.156	−0.066	−0.026	0.046	0.076	0.158	0.039	0.094	0.076	0.103	0.158	0.088
Embarrassment	−0.180*	−0.062	−0.215**	−0.080	−0.068	−0.162*	0.253**	0.145	0.257**	−0.029	0.081	0.079	0.033	0.259**	0.057
Stoma care problems	0.109	0.245	0.195	0.000	−0.140	0.100	0.023	0.349	0.196	0.185	−0.179	0.049	0.099	0.011	−0.358
Impotence	−0.172	−0.179	−0.163	−0.126	−0.135	−0.198	0.152	−0.082	0.239*	−0.002	0.101	−0.042	0.080	0.100	0.156
Dyspareunia	−0.032	−0.193	0.041	−0.324**	−0.093	−0.134	0.021	0.023	0.117	−0.066	0.001	0.020	−0.016	0.091	0.129
Anxiety	0.278**	0.229**	0.225**	0.499**	0.206*	0.287**	−0.292**	−0.118	−0.253**	−0.219**	−0.157	−0.191	−0.026	−0.067	−0.261
Weight	0.229**	0.351**	0.248**	0.254**	0.150	0.185*	−0.294**	−0.168*	−0.288**	−0.084	−0.021	−0.320**	−0.095	−0.003	−0.043
Sexual interest (men)	0.181	0.204	0.339**	0.170	0.243*	0.118	−0.259*	0.061	−0.163	0.011	−0.346**	−0.225*	−0.176	−0.004	0.012
Sexual interest (women)	−0.132	0.199	0.187	−0.135	−0.006	0.035	−0.082	−0.051	0.116	−0.100	0.039	−0.086	0.012	−0.027	−0.009

** Correlation is significant at the 0.01 level, *Correlation is significant at the 0.05 level, QL – quality of life, PF – physical functioning, RF – role functioning, EF – emotional functioning, CF – cognitive functioning, SF – social functioning, FA – fatigue, NV – nausea and vomiting, PA – pain, DY – dyspnea, SL – insomnia, AP – appetite loss, CO – constipation, DI – diarrhea, FI – financial difficulties.

## Known-groups validity

The scales and single-items of CR-29 questionnaire based on tumor location, stoma presence, and neoadjuvant therapy are shown in Table 5. Regarding tumor location (Colon vs. Rectum) there were significant differences in a few areas: Blood/mucus in stool and stool frequency were more problematic in rectal cancer patients, with *p*-values of 0.046 and 0.029, respectively. Abdominal pain and dry mouth were significantly higher in colon cancer patients (*p* = 0.016, and *p* = 0.013). Dyspareunia showed a significant difference (*p* = 0.006), with higher scores in rectal cancer patients. Urinary incontinence and embarrassment were significantly more common in patients with a stoma (*p* = 0.040, *p* < 0.001). Hair loss and embarrassment were significantly more common in patients who received neoadjuvant therapy, with *p*-values of 0.026 and 0.048, respectively while dry mouth was more common in patients who did not receive neoadjuvant therapy (*p* = 0.015).

**Table 5.** Known-groups validity of EORTC QLQ-CR29 questionnaire

	Tumor location			Stoma			Neoadjuvant therapy		
CR-29 scales	Colon (n = 76)	Rectum (n = 73)	*p*	Yes (n = 16)	No (n = 134)	*p*	Yes (n = 39)	No (n = 111)	*p*
Urinary frequency	32.9 ± 23.7	35.8 ± 24.5	0.456	39.6 ± 22.7	33.8 ± 24.2	0.367	39.3 ± 23.4	32.7 ± 24.1	0.141
Blood and mucus in stool	20.0 ± 24.5	27.2 ± 26.1	**0.046**	17.7 ± 20.6	24.5 ± 26.1	0.386	21.8 ± 20.6	24.5 ± 27.2	0.996
Stool frequency	12.9 ± 23.5	17.6 ± 21.1	**0.029**	18.7 ± 18.1	15.2 ± 23.2	0.178	15.8 ± 17.5	15.5 ± 24.4	0.283
Body image	81.0 ± 24.9	78.2 ± 24.2	0.494	74.3 ± 25.2	80.2 ± 24.4	0.264	74.4 ± 25.1	81.4 ± 24.1	0.123
CR-29 single items									
Urinary incontinence	8.8 ± 17.5	7.3 ± 20.2	0.231	16.7 ± 24.3	7.2 ± 18.0	**0.040**	12.0 ± 25.9	6.9 ± 15.6	0.555
Dysuria	5.7 ± 16.7	4.1 ± 13.5	0.504	10.4 ± 20.1	4.2 ± 14.4	0.069	6.8 ± 17.4	4.2 ± 14.3	0.340
Abdominal pain	26.7 ± 28.8	16.9 ± 26.7	**0.016**	25.0 ± 35.5	21.9 ± 27.4	0.973	17.9 ± 28.4	23.7 ± 28.2	0.172
Buttock pain	14.5 ± 22.0	18.7 ± 25.4	0.307	27.1 ± 34.9	15.4 ± 21.9	0.217	23.1 ± 28.8	14.4 ± 21.4	0.097
Bloating	28.5 ± 27.6	22.8 ± 24.1	0.244	29.2 ± 29.5	25.6 ± 25.8	0.657	23.1 ± 24.4	27.0 ± 26.8	0.479
Dry mouth	28.9 ± 25.7	19.2 ± 24.2	**0.013**	18.7 ± 21.0	24.9 ± 25.7	0.428	16.2 ± 22.8	27.0 ± 25.6	**0.015**
Hair loss	3.5 ± 10.3	9.6 ± 23.2	0.209	12.5 ± 24.0	5.7 ± 17.1	0.140	12.8 ± 24.9	4.2 ± 14.3	**0.026**
Taste	7.5 ± 19.3	4.1 ± 13.5	0.251	4.2 ± 11.4	6.0 ± 17.1	0.933	4.3 ± 13.6	6.3 ± 17.7	0.585
Flatulence	20.6 ± 29.8	21.5 ± 28.5	0.729	22.9 ± 26.4	21.1 ± 29.6	0.603	23.1 ± 27.7	20.7 ± 29.8	0.450
Fecal incontinence	12.3 ± 24.2	10.5 ± 22.1	0.737	8.3 ± 19.2	12.2 ± 24.0	0.558	10.3 ± 21.8	12.3 ± 24.2	0.727
Sore skin	11.4 ± 20.0	10.5 ± 17.5	0.941	6.2 ± 13.4	11.9 ± 19.8	0.297	9.4 ± 18.6	12.0 ± 19.5	0.368
Embarrassment	7.0 ± 22.6	18.7 ± 27.2	**<0.001**	31.2 ± 31.0	10.7 ± 24.0	**<0.001**	16.2 ± 22.8	11.7 ± 26.5	**0.048**
Stoma care problems	33.3 ± 0.0	15.4 ± 22.0	0.113	18.7 ± 21.0	/	/	12.1 ± 16.8	33.3 ± 23.6	0.075
Impotence	31.6 ± 32.8	26.2 ± 26.1	0.590	29.2 ± 21.4	28.7 ± 30.3	0.765	28.0 ± 24.9	29.1 ± 31.5	0.890
Dyspareunia	4.4 ± 13.8	18.3 ± 28.3	**0.006**	8.3 ± 15.4	10.7 ± 23.2	0.960	14.3 ± 28.4	9.5 ± 20.8	0.561
Anxiety	45.2 ± 31.6	44.3 ± 29.9	0.917	37.5 ± 29.5	45.8 ± 30.8	0.306	44.4 ± 28.9	45.0 ± 31.3	0.917
Weight	74.1 ± 27.0	68.0 ± 31.1	0.273	68.7 ± 33.3	71.4 ± 28.7	0.871	69.2 ± 31.9	71.8 ± 28.1	0.640
Sexual interest (men)	39.5 ± 28.8	43.6 ± 28.0	0.513	37.5 ± 11.8	42.1 ± 29.6	0.698	38.7 ± 20.8	43.0 ± 31.2	0.542
Sexual interest (women)	9.6 ± 18.8	8.6 ± 17.1	0.883	8.3 ± 15.4	9.1 ± 18.3	0.940	4.8 ± 12.1	10.1 ± 19.0	0.366

## Discussion

Considering that QoL assessments have become integral to the comprehensive management of cancer patients, offering indispensable insights into the disease and treatment impacts from the patient’s perspective. Our study aimed to translate, culturally adapt, and psychometrically validate the EORTC QLQ-CR29 questionnaire for Serbian colorectal cancer patients. This effort is particularly relevant given the rising incidence of colorectal cancer (Cancer Registry 2023; Morgan et al. 2023).

The absence of missing data underscores a high level of participant compliance, suggesting that the questionnaire was neither difficult, confusing, nor burdensome for the patients. This implies a strong acceptance of the questionnaire among Serbian patients. In terms of construct validity, our findings revealed that the Cronbach’s alpha coefficients for urinary frequency, blood and mucus in stool, and stool frequency scales were 0.769, 0.802, and 0.810, respectively which indicate good internal consistency. This shows that the items within these scales are cohesively measuring the intended constructs among patients. Furthermore, the body image scale, with a Cronbach’s alpha of 0.855, exhibits excellent internal consistency. Compared to the original study, our Cronbach’s alpha values were higher for all scales (Whistance et al. 2009). Moreover, when compared to the Spanish, Dutch, Chinese, Malaysian, Moroccan, Ethiopian, Mexican and Polish versions, our study reported higher Cronbach’s alpha coefficients across all scales (Abebe et al. 2021; Arrarás et al. 2011; Hernández-Marín et al. 2024; Lin et al. 2017; Magaji et al. 2015; Sanna et al. 2017; Stiggelbout et al. 2016; Yacir et al. 2022). Studies by Ihn et al. (2015) and Shen et al. (2018) presented Cronbach’s alpha values similar to ours, indicating a consistent measure of internal consistency across different populations and settings.

For patients without a stoma, the internal consistency across all scales being above the 0.7 threshold (range, 0.764–0.871) highlights the questionnaire’s robustness in this subgroup. However, in patients with a stoma, while the urinary frequency and body image scales show strong internal consistency (alpha coefficients greater than 0.8), the blood/mucus in stool scale shows a lower reliability (alpha = 0.654), and the stool frequency scale exhibits poor reliability (alpha = 0.225). Other studies also showed better internal consistency of these scales among patients without stoma (Lin et al. 2017; Magaji et al. 2015; Yacir et al. 2022). In comparison with the original study, we found that the internal consistency of CR29 scales was better in patients without stoma. However, in patients with stoma, Cronbach’s alpha coefficients were slightly lower for scales blood/mucus in stool and especially for stool frequency (Whistance et al. 2009). Lin et al. (2017) and Magaji et al. (2015) also revealed the lowest Cronbach’s alpha coefficient value in stool frequency among patients with stoma. These findings suggest the need for cautious interpretation of scores from these 2 scales in patients with a stoma.

The split-half reliability in our study was 0.872. Our study demonstrated that the EORTC QLQ-CR29 is a reliable tool for assessing quality of life in patients with colorectal cancer, providing stable and consistent results across its scales. This outcome aligns with data from other studies that have also confirmed the CR29 questionnaire’s reliability (Arrarás et al. 2011; Ihn et al. 2015; Lin et al. 2017; Shen et al. 2018; Whistance et al. 2009; Wickramasinghe et al. 2020). In research contexts, the high reliability of the EORTC QLQ-CR29 supports its use in longitudinal studies to assess changes over time, as well as in intervention studies to evaluate treatment outcomes. This reinforces the questionnaire’s value in both clinical practice and research, providing reliable insights into patients’ quality of life.

The Serbian CR29 questionnaire demonstrated strong convergent validity, indicating that the scales are effective in measuring related constructs, aligning with the original study (Whistance et al. 2009). Divergent validity was mostly satisfactory, with items showing low or no correlation with unrelated scales, ensuring a comprehensive and multi-dimensional QoL assessment without overlap. Exceptions were noted in stool frequency and body image scales among stoma patients, which was similar to divergent validity challenges in Polish, Korean, Moroccan, and Chinese versions (Ihn et al. 2015; Lin et al. 2017; Sanna et al. 2017; Yacir et al. 2022). Overall, the Serbian CR29’s construct validity is very good.

The significant correlations, both positive and negative, across various scales and items between the CR29 and C30 questionnaires indicate that the CR29 is effectively capturing aspects of health status and quality of life that align with the broader and well-established QLQ-C30. Scales and items with closely related content exhibit higher correlation coefficients, aligning with findings from previous research (Abebe et al. 2021; Ihn et al. 2015; Sanna et al. 2017). Additionally, the presence of scales and items with low correlation coefficients suggests that the CR29 and C30 questionnaires are measuring distinct concepts, highlighting the complementary nature of the 2 instruments (Abebe et al. 2021; Lin et al. 2017; Sanna et al. 2017). Consequently, to ensure a comprehensive assessment of quality of life in patients with colorectal cancer, the CR29 module should be used together with the core C30 questionnaire.

The CR29 questionnaire effectively differentiates QoL issues in colorectal cancer patients based on tumor location, stoma presence, and neoadjuvant therapy. Rectal cancer patients reported more issues with blood/mucus in stool and stool frequency, whereas colon cancer patients experienced more abdominal pain and dry mouth, linked to colon blockage and stress-related symptoms (Acevedo-Ibarra et al. 2021; Antoniadis et al. 2024). Additionally, the significantly higher rates of dyspareunia in rectal cancer patients could be attributed to the proximity to sexual organs, impacting sexual function (Ihn et al. 2015; Kowal et al. 2022; Reese et al. 2018). Stoma-associated embarrassment and psychological burden are significant, with rectal cancer patients often experiencing worse body image and urinary incontinence issues, although body image differences were not statistically significant (Abebe et al. 2021; Bachri et al. 2023; Ihn et al. 2015; Lin et al. 2017; Sanna et al. 2017; Whistance et al. 2009). Chemotherapy, a common component of neoadjuvant therapy for colorectal cancer, can cause hair loss (Saraswat et al. 2019). This side effect can be particularly distressing for patients, as hair is often associated with personal identity and social perceptions of health (Özüsağlam and Can 2021; Mao et al. 2019). Studies showed that hair loss is more common in patients who received neoadjuvant therapy (Ihn et al. 2015; Shen et al. 2018).

This study has several strengths, including a thorough validation process and high participant compliance. The study’s limitations are primarily due to its single-center design, which might restrict the generalizability of the findings. However, it’s important to note that the Clinic for Digestive Surgery at the University Clinical Center of Serbia, is the leading and the largest center for colorectal cancer treatment in the country. This center draws patients from diverse regions and socio-economic backgrounds across Serbia, somewhat mitigating concerns about representativeness. Other limitations are the relatively small sample size for specific subgroups, such as patients with a stoma which might have affected results in this subgroup.

The QLQ-CR29 questionnaire has been skillfully translated, adapted to the cultural context and rigorously validated though psychometric methods for Serbian patients with colorectal cancer. The findings underscore the questionnaire’s reliability and validity across various areas. The high level of participant compliance suggests the questionnaire is well-accepted among Serbian patients, indicating its appropriateness and non-burdensome nature. This validated tool enables healthcare professionals to comprehensively assess and monitor the quality of life impacts specific to colorectal cancer.
